# Prevalence of patient-reported and clinician-graded cognitive symptomatic adverse events in older patients with advanced cancer

**DOI:** 10.1093/jncics/pkag086

**Published:** 2026-08-24

**Authors:** Riham Alieldin, Mostafa Mohamed, Chin-Shang Li, Michelle C Janelsins, Rachael Tylock, Karen M Mustian, Luke Peppone, Charles Kamen, Po-Ju Lin, Kah Poh Loh, AnnaLynn M Williams, Brian J Altman, Paula M Vertino, Hongying Sun, Umang Gada, Supriya G Mohile, Judith O Hopkins, Bryan A Faller, Vincent Vinciguerra, Allison Magnuson

**Affiliations:** Department of Surgery, Division of Supportive Care in Cancer, University of Rochester, Rochester, NY, United States; Department of Public Health Medicine, University of Rochester, Rochester, NY, United States; Wilmot Cancer Institute, University of Rochester, Rochester, NY, United States; Department of Surgery, Division of Supportive Care in Cancer, University of Rochester, Rochester, NY, United States; Department of Surgery, Division of Supportive Care in Cancer, University of Rochester, Rochester, NY, United States; Wilmot Cancer Institute, University of Rochester, Rochester, NY, United States; Wilmot Cancer Institute, University of Rochester, Rochester, NY, United States; Department of Surgery, Division of Supportive Care in Cancer, University of Rochester, Rochester, NY, United States; Wilmot Cancer Institute, University of Rochester, Rochester, NY, United States; Department of Surgery, Division of Supportive Care in Cancer, University of Rochester, Rochester, NY, United States; Wilmot Cancer Institute, University of Rochester, Rochester, NY, United States; Department of Surgery, Division of Supportive Care in Cancer, University of Rochester, Rochester, NY, United States; Wilmot Cancer Institute, University of Rochester, Rochester, NY, United States; Department of Surgery, Division of Supportive Care in Cancer, University of Rochester, Rochester, NY, United States; Wilmot Cancer Institute, University of Rochester, Rochester, NY, United States; Wilmot Cancer Institute, University of Rochester, Rochester, NY, United States; Department of Medicine, Division of Hematology/Oncology, University of Rochester, Rochester, NY, United States; Department of Surgery, Division of Supportive Care in Cancer, University of Rochester, Rochester, NY, United States; Department of Public Health Medicine, University of Rochester, Rochester, NY, United States; Wilmot Cancer Institute, University of Rochester, Rochester, NY, United States; Wilmot Cancer Institute, University of Rochester, Rochester, NY, United States; Department of Biomedical Genetics, University of Rochester, Rochester, NY, United States; Wilmot Cancer Institute, University of Rochester, Rochester, NY, United States; Department of Biomedical Genetics, University of Rochester, Rochester, NY, United States; Department of Surgery, Division of Supportive Care in Cancer, University of Rochester, Rochester, NY, United States; Department of Surgery, Division of Supportive Care in Cancer, University of Rochester, Rochester, NY, United States; Wilmot Cancer Institute, University of Rochester, Rochester, NY, United States; Department of Medicine, Division of Hematology/Oncology, University of Rochester, Rochester, NY, United States; Southeast Clinical Oncology Research Consortium (SCOR), Novant Health Cancer Institute, Winston-Salem, NC, United States; Heartland Cancer Research NCORP (HEARTLAND), Saint Louis, MO, United States; Northwell Health NCORP (NORTHWELL), Lake Success, NY, United States; Wilmot Cancer Institute, University of Rochester, Rochester, NY, United States; Department of Medicine, Division of Hematology/Oncology, University of Rochester, Rochester, NY, United States

## Abstract

**Purpose:**

Cancer-related cognitive impairment is common in patients receiving cancer treatment but may be underdetected by clinician-graded adverse events (AEs) alone. Patient-reported outcomes and cognitive screening may improve identification of cognitive symptoms.

**Methods:**

We conducted a secondary analysis of the nationwide, multicenter GAP70+ trial of adults aged ≥70 years with advanced cancer starting systemic therapy. Cognitive symptoms were assessed longitudinally using patient-reported and clinician-graded cognitive AEs, and Mini-Cog screening at baseline, 4-6 weeks, 3 months, and 6 months. We examined prevalence, longitudinal trajectories, and associations with Mini-Cog impairment. Statistical significance was set at 2-sided *P *< .05.

**Results:**

Among 704 participants (mean age = 77.2 years; range = 70-96 years), patient-reported cognitive AEs were more prevalent than clinician-graded cognitive AEs at all time points: 19% vs 0.48% at 4-6 weeks, 18% vs 2.5% at 3 months, and 22% vs 0.44% at 6 months (all *P* < .001). Patient-reported cognitive AEs also fluctuated within patients over time. Impaired Mini-Cog was associated with higher patient-reported cognitive AEs at all post-baseline time points: 31% vs 15% at 4-6 weeks, 34% vs 14% at 3 months, and 49% vs 15% at 6 months (all *P* < .001). In contrast, associations with clinician-graded cognitive AEs were observed only at 3 months (*P* = .004) and 6 months (*P* = .04).

**Conclusions:**

Cognitive symptomatic AEs are common and often underdetected by clinician grading alone in older adults with advanced cancer. Combining patient-reported AEs with brief cognitive screening may improve detection during treatment.

## Introduction

Cancer-related cognitive impairment (CRCI) is increasingly recognized as a prevalent side effect in older adults undergoing cancer therapy.^1-5^ Studies indicate that up to 80% of patients with cancer experience some degree of cognitive decline during chemotherapy,^1^^,^^4-8^ which can impact their overall quality of life.^1^^,^^3^^,^^9^^,^^10^ Cognitive decline in this population is multifactorial. Some older adults begin cancer treatment with pre-existing cognitive impairment or lower cognitive reserve,^11-13^ and cancer-related factors such as psychological distress, systemic inflammation, and disease burden may contribute to cognitive symptoms before treatment.^3^^,^^14^^,^^15^

Estimates suggest that 3%-7% of older adults with cancer have dementia, with many more experiencing mild cognitive impairment or subtle decline.^11^^,^^13^ Cancer treatment may further exacerbate pre-existing impairment or induce treatment-related neurotoxicity, resulting in CRCI.^3^^,^^12^^,^^13^^,^^16-18^ However, routine cognitive assessment in oncology practice remains inconsistent,^19^ limiting early identification of patients at risk for cognitive symptomatic adverse events (AEs).

Cognitive assessment in older adults with cancer is clinically important.^13^ Baseline cognition can inform treatment decision making, consent, and adherence,^20^^,^^21^ whereas serial monitoring can detect emerging cognitive symptomatic AEs that may otherwise be attributed to aging or comorbidities.^6^^,^^13^^,^^22^^,^^23^ Early recognition can guide supportive interventions and help preserve function, quality of life, and independence in older adults.^13^^,^^22^^,^^24^^,^^25^

Despite the high prevalence of CRCI, cognitive symptoms are often underrecognized when relying solely on clinician assessment.^26^^,^^27^ Traditional AE reporting in oncology trials uses the clinician-graded Common Terminology Criteria for Adverse Events (CTCAE).^26^^,^^27^ However, treatment tolerability reflects patients’ lived experiences of symptoms,^28^ prompting the development of patient-reported measures. The Patient-Reported Outcomes version of the CTCAE (PRO-CTCAE) was designed to capture symptom severity, frequency, and interference directly from the patient perspective complementing clinician grading and providing a more comprehensive assessment of symptomatic AEs, including cognitive symptoms that may otherwise be overlooked.^26^^,^^27^^,^^29^^,^^30^

Prior studies in older adults with cancer have demonstrated substantial discordance between clinician-graded CTCAE and PRO-CTCAE reporting.^31^^,^^32^ In the same dataset, clinician-graded CTCAE captured grade ≥2 pain in fewer than 25% of patients reporting moderate or worse pain, underscoring underdetection with traditional AE reporting. However, concordance for cognitive AEs remains unknown, representing an important gap in geriatric oncology.^31^

In this study, we investigate the prevalence and longitudinal trajectories of cognitive symptomatic AEs in older adults with advanced cancer by comparing patient-reported PRO-CTCAE with clinician-graded CTCAE. We further examine the association of a brief cognitive screening tool (Mini-Cog) with these 2 reporting modalities over time. Through this comparative approach, we aim to clarify patterns of cognitive AE detection and inform strategies for cognitive assessment and toxicity monitoring in older adults with cancer.

## Methods

### Study design

This study was a secondary analysis of data from the nationwide, multicenter, cluster-randomized geriatric assessment (GA) for patients 70 years and older trial, GAP70+; ClinicalTrials.gov identifier: NCT02054741. The primary study evaluated whether providing GA information to community oncologists reduced clinician-rated grade 3-5 CTCAE toxicities in older adults with advanced cancer initiating a new systemic therapy.^24^ It was conducted through the University of Rochester Cancer Center National Cancer Institute Community Oncology Research Program (URCC NCORP) Research Base and was approved by institutional review boards at participating sites. All participants provided written informed consent.

Eligible patients for the primary study were (1) aged 70 years or older; (2) diagnosed with incurable, stage III/IV solid tumor or lymphoma; (3) impaired in at least one GA domain, excluding the polypharmacy domain; and (4) planning to start a new cancer treatment regimen with a high risk of grade 3-5 AEs based on CTCAE, version 4. Eligible regimens were determined at the discretion of the enrolling physicians and reviewed by blinded clinical staff at the URCC NCORP Research Base. All participants were enrolled between July 2014 and March 2019. The analytic sample for this secondary analysis included 704 participants from the GAP70+ trial who completed baseline assessments and at least 1 follow-up assessment of cognitive outcomes.

### Measures

For this analysis, cognitive data were derived from 3 complementary sources within the GAP70+ study: (1) patient-reported cognitive symptomatic AEs assessed using the PRO-CTCAE^27^^,^^29^^,^^30^ memory and concentration items, (2) clinician-graded cognitive AEs assessed using CTCAE version 4.0,^26^^,^^27^ and (3) objective cognitive assessment measured using the Mini-Cog. To facilitate comparison across modalities, we refer to patient-reported cognitive symptomatic AEs as **Cog-PRO** (patient-reported cognitive outcomes) and clinician-graded cognitive symptomatic AEs as **Cog-CTCAE** (clinician-graded cognitive adverse events) throughout the article.

Cognitive data were collected longitudinally across 4 study time points: baseline (A1), 4-6 weeks (A2), 3 months (A3), and 6 months (A4) after baseline assessment. PRO-CTCAE and Mini-Cog were collected at all time points, whereas CTCAE data were collected starting at 4-6 weeks. This allowed evaluation of the prevalence and trajectories of cognitive AEs and the associations between patient-reported, clinician-graded, and objective (Mini-Cog) cognitive assessments over time.

Additional baseline demographic, clinical, and geriatric assessment variables collected in the parent GAP70+ study are summarized in Table S4 and detailed previously in the parent trial.^24^

### Cog-PRO

From the GAP70+ PRO-CTCAE dataset, we included cognitive-related AE items, which included (1) What was the severity of your problems with concentration at its worst? and (2) What was the severity of your problems with memory at its worst?^29^^,^^33^ Both items included severity and interference components. Severity was rated on a 5-point scale (none, mild, moderate, severe, very severe), and interference on a 5-point scale (not at all, a little bit, somewhat, quite a bit, very much). For analysis, these variables were dichotomized into categories of none or mild vs moderate or worse for the severity component, and not at all vs any interference.

### Cog-CTCAE

Cog-CTCAE derived from CTCAE data collected in the parent GAP70+ trial^24^ using NCI CTCAE version 4.^26^^,^^34^ CTCAE adverse events were assessed by trained clinicians and coordinators at participating community oncology sites using standardized CTCAE training materials and NCI adverse event reporting instructions. Study coordinators prospectively captured grade 3-5 toxicities, confirmed grading with patients and treating oncologists, and medical records were centrally reviewed by a Research Base team masked to study group.^24^ For this secondary analysis, we reviewed all clinician-reported CTCAE AEs at the 4-6 weeks assessment and identified 6 potential AE criteria related to cognition (cognitive disturbance, concentration impairment, memory impairment, delirium, decreased level of consciousness, and confusion). The selection of these cognitive-related AE codes was independently conducted by 2 clinicians (R.A. and M.M.) and verified by a third reviewer (S.G.M.) to ensure consistency. Each event was graded according to CTCAE v4^26^^,^^34^ criteria and analyzed both as a binary variable (present vs absent) and as a dichotomized variable representing low grade (≤1) vs clinically significant (≥2) impairment.^22^^,^^35^

### Mini-Cog (objective cognitive screening)

Objective cognitive function was assessed using the Mini-Cog screening test,^36^^,^^37^ which includes 2 standard tasks: 3-word recall and clock drawing. The Mini-Cog was analyzed as a binary variable (impaired screening result vs not impaired), based on established scoring criteria.^36^^,^^37^

### Statistical analysis

Baseline characteristics were summarized using descriptive statistics, reported as means (SD) for continuous variables and frequencies (%) for categorical variables. Cognitive symptomatic AEs were assessed at each time point using patient-reported PRO-CTCAE (Cog-PRO) and clinician-graded CTCAE (Cog-CTCAE). Prevalence estimates with 95% confidence intervals (CIs) were calculated and displayed graphically to compare reporting modalities, with paired postbaseline comparisons evaluated using McNemar tests. Longitudinal trajectories of patient-reported concentration and memory symptom severity were visualized by using Sankey diagrams.

Associations between Mini-Cog impairment (impaired vs not-impaired) and Cog-PRO and Cog-CTCAE were assessed at each postbaseline time point using chi-square (χ²) or Fisher exact tests. Longitudinal changes in PRO-CTCAE cognitive symptomatic AEs were evaluated using generalized estimating equations with a binomial distribution, logit link, and exchangeable correlation structure, with Sidak adjustment for multiple comparisons. Statistical significance was defined as 2-sided *P *< .05. All analyses were performed using SAS version 9.4 (SAS Institute).

## Results

### Sample characteristics

A total of 704 patients were included. The mean age was 77.21 years (SD = 5.45; range = 70-96). Most participants were male (57%) and non-Hispanic White (88%). The predominant cancer types were gastrointestinal (35%) and lung (25%), and 87% had stage IV disease. Additional data on patients’ characteristics and baseline geriatric assessments are reported in Table 1 and Table S4.

**Table 1.** Study population characteristics, overall, and by pretreatment cognitive impairment (impaired Mini-Cog status) (*N* = 704).

Characteristic	Overall (*N* = 704)	Impaired Mini-Cog (*n* = 248)	Non-impaired Mini-Cog (*n* = 456)	*P*
Age mean ± SD	77.21 **±** 5.45	78.16 ± 5.51	76.70 ± 5.35	<.001^b^
	***n* (%)**			
Age group				.03^b^
70-79	484 (69)	158 (64)	326 (71)	
80-89	202 (29)	82 (33)	120 (26)	
≥90	18 (2.6)	8 (3.2)	10 (2.2)	
Gender				.5^a^
Male	399 (57)	145 (58)	254 (56)	
Female	305 (43)	103 (42)	202 (44)	
Race				<**.001**^a^
Non-Hispanic White	618 (88)	201 (81)	417 (92)	
Black	51 (7.2)	33 (13)	18 (4.0)	
Other	34 (4.8)	14 (5.7)	20 (4.4)	
Missing	1	0	1	
Education level				<**.001**^b^
Less than high school	109 (15)	65 (26)	44 (9.7)	
High school graduate	238 (34)	88 (35)	150 (33)	
Some college or above	357 (51)	95 (38)	262 (57)	
Income level				**<.001** ^a^
Less than or equal to $50k	361 (51)	140 (56)	221 (48)	
More than $50k	189 (27)	44 (18)	145 (32)	
Declined to answer	154 (22)	64 (26)	90 (20)	
Marital status				.6^a^
Single, never married	15 (2.1)	6 (2.4)	9 (2.0)	
Married or domestic partnership	442 (63)	150 (60)	292 (64)	
Separated, widowed, or divorced	247 (35)	92 (37)	155 (34)	
Cancer type				1^a^
Breast	55 (7.8)	18 (7.3)	37 (8.1)	
Gastrointestinal	243 (35)	85 (34)	158 (35)	
Genitourinary	109 (15)	39 (16)	70 (15)	
Gynecological	41 (5.8)	15 (6.1)	26 (5.7)	
Lung	175 (25)	60 (24)	115 (25)	
Lymphoma	46 (6.5)	19 (7.7)	27 (5.9)	
Other	35 (5.0)	12 (4.8)	23 (5.0)	
Cancer stage				.7^a^
III	76 (11)	27 (11)	49 (11)	
IV	615 (87)	215 (87)	400 (88)	
Others	13 (1.9)	6 (2.4)	7 (1.5)	
Treatment type				**.03** ^a^
Single chemotherapy	145 (21)	43 (17)	102 (22)	
Multiple chemo	326 (46)	108 (44)	218 (48)	
Chemo + other agents	149 (21)	57 (23)	92 (20)	
Other agents	84 (12)	40 (16)	44 (9.7)	

a χ² test.

b Wilcoxon rank-sum test.

### Prevalence of cognitive symptomatic adverse events

**Figure 1.**
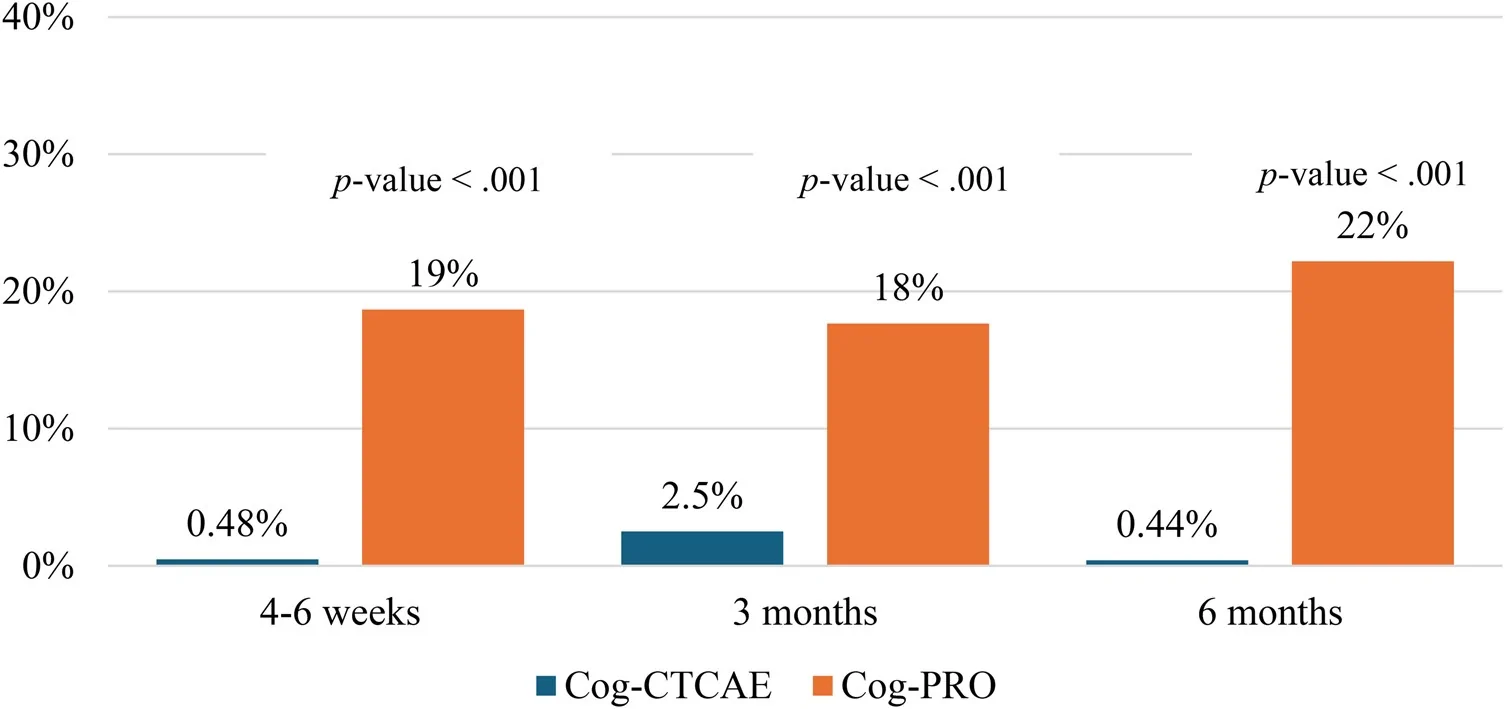
Prevalence of cognitive symptomatic adverse events using Cog-CTCAE (grade 2+) and Cog-PRO (moderate or greater) across post-baseline time points. Prevalence of clinician-graded cognitive symptomatic adverse events, defined as Cog-CTCAE grade 2 or higher, and patient-reported cognitive symptomatic adverse events, defined as Cog-PRO moderate or greater, at 4-6 weeks, 3 months, and 6 months after baseline. Paired binary data comparing Cog-CTCAE and Cog-PRO at each postbaseline time point were analyzed using McNemar’s test. AE = adverse event; Cog-CTCAE = clinician-graded cognitive adverse event based on the Common Terminology Criteria for Adverse Events; Cog-PRO = patient-reported cognitive symptomatic adverse event; PRO = patient-reported outcome.

The prevalence of cognitive symptomatic AEs was consistently higher when assessed by patients using the PRO-CTCAE (Cog-PRO) compared with clinician-graded CTCAE (Cog-CTCAE) across all time points (Figure 1).

At 4-6 weeks, 19% of patients reported moderate or greater cognitive symptomatic AEs on the PRO-CTCAE compared with only 0.48% with grade ≥2 cognitive symptomatic AEs on the clinician-graded CTCAE (*P* < .001). At 3 months, prevalence was 18% vs 2.5% (*P* < .001), and at 6 months, 22% vs 0.44%, respectively (*P* < .001) (Figure 1 and Table S1). Patient-reported cognitive symptoms were consistently more frequent than clinician-graded AEs at all post-baseline assessments (all *P* < .001). Additional prevalence estimates for all cognitive measures across assessment time points are provided in Tables S1 and S2.

### Trajectories of patient-reported cognitive symptomatic adverse events

**Figure 2.**
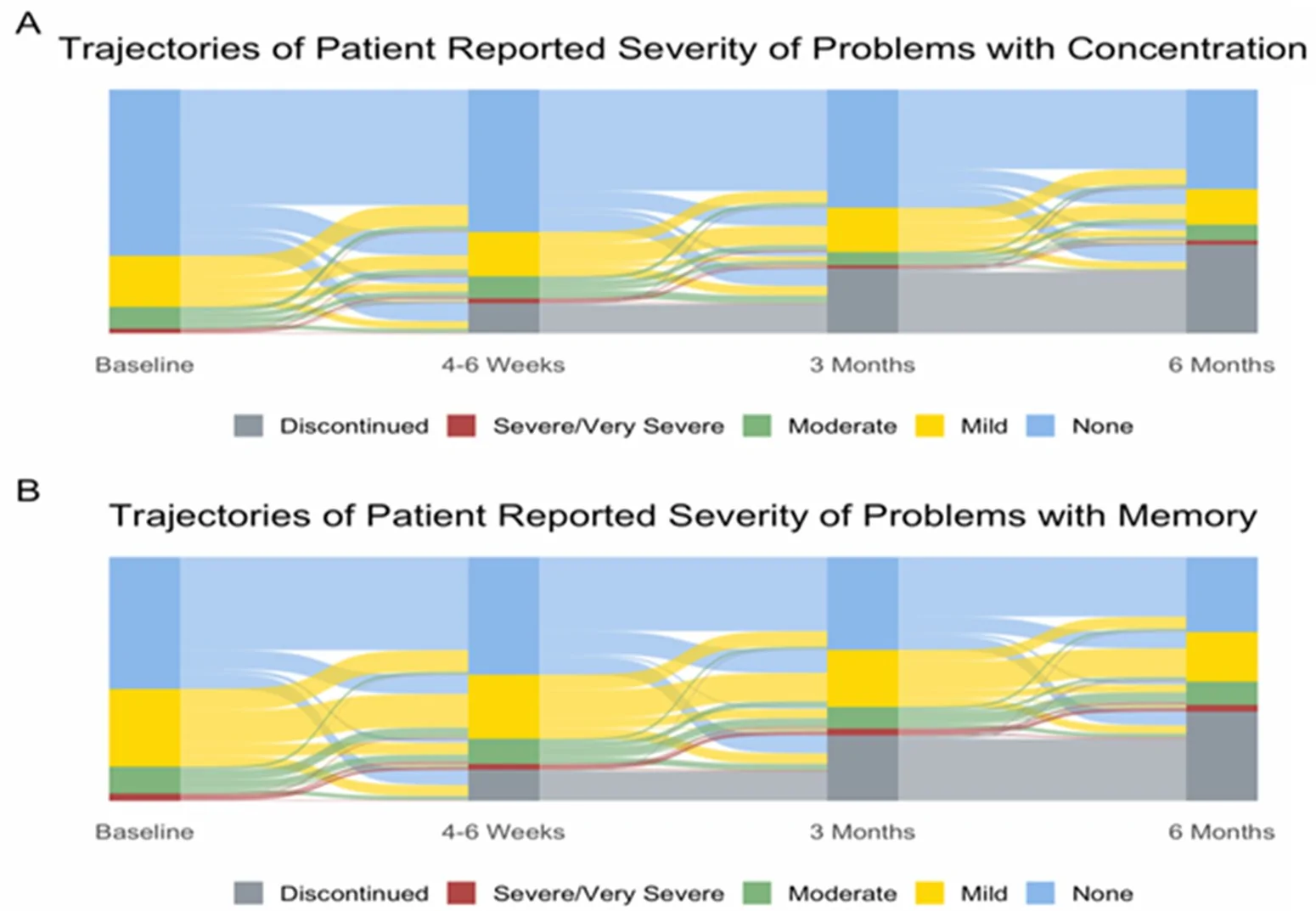
Trajectories of patient-reported concentration and memory problems over time. Sankey diagrams showing longitudinal trajectories of patient-reported severity of problems with concentration (A) and problems with memory (B) from baseline to 4-6 weeks, 3 months, and 6 months. Flow widths represent the proportion of patients transitioning between symptom severity categories over time. Symptom categories include none, mild, moderate, severe, or very severe, and discontinued. PRO = patient-reported outcome.

Longitudinal assessment of patient-reported concentration and memory cognitive symptomatic AEs demonstrated within-patient variability over time (Figure 2). While most patients reported stable or mild symptoms, a subset transitioned between none/mild and moderate or severe categories, indicating dynamic cognitive symptom patterns during treatment.

In the GEE model, the odds of reporting patient-reported cognitive symptoms (Cog-PRO) at 6 months (A4) were higher compared with baseline (A1) (odds ratio [OR] = 1.42, 95% CI = 1.14 to 1.77; *P* = .002). Similarly, the odds of reporting cognitive symptomatic AEs at 6 months were higher compared with 4-6 weeks (OR = 1.38, 95% CI = 1.10 to 1.72; *P* = .005) and compared with 3 months (OR = 1.36, 95% CI = 1.11 to 1.66; *P* = .003). Changes between other time point comparisons are presented in Table S3.

### Association between objective cognitive impairment and cognitive symptomatic AEs

Impaired Mini-Cog performance was statistically significantly associated with both clinician-graded (Cog-CTCAE) and patient-reported (Cog-PRO) cognitive symptomatic AEs across multiple time points. At 4-6 weeks, 31% of patients with abnormal Mini-Cog reported moderate or greater cognitive symptomatic AEs on the Cog-PRO compared with 15% of those with normal Mini-Cog scores (*P *< .001) (Table 2). The association between impaired Mini-Cog and clinician-graded CTCAE cognitive AEs was not statistically significant at 4-6 weeks (*P *= .5). At 3 months, impaired Mini-Cog was significantly associated with both clinician-graded and patient-reported cognitive symptomatic AEs (*P *= .004 and *P *< .001, respectively). Specifically, 34% of those with impaired Mini-Cog vs 14% of those with no impaired Mini-Cog reported moderate or greater symptoms. A similar pattern persisted at 6 months (*P *= .04 for Cog-CTCAE and *P *< .001 for Cog-PRO), where 49% of patients with impaired Mini-Cog reported moderate or greater cognitive symptoms, compared with 15% of patients without Mini-Cog impairment (Table 2).

**Table 2.** Association between Mini-Cog impairment and cognitive symptomatic adverse events.

*N* = 704		Cog-CTCAE (grade 2+)			Cog-PRO (moderate or greater)		
Assessment time point	Mini-Cog impairment	No (%)	Yes (%)	*P*	No (%)	Yes (%)	*P*
4-6 weeks	No	485 (100)	2 (0.41)	.5^a^	413 (85)	75 (15)	<**.001**^b^
	Yes	123 (99)	1 (0.81)		86 (69)	38 (31)	
3 months	No	417 (99)	6 (1.4)	**.004** ^b^	361 (86)	57 (14)	<**.001**^b^
	Yes	91 (93)	7 (7.1)		64 (66)	33 (34)	
6 months	No	369 (100)	0 (0)	**.04** ^a^	313 (85)	55 (15)	<**.001**^b^
	Yes	85 (98)	2 (2.3)		44 (51)	42 (49)	

Abbreviations: Cog-CTCAE = clinician-graded cognitive adverse event; Cog-PRO = patient-reported cognitive symptomatic adverse event. Percentages are row percentages. *P*-values were calculated using Fisher’s exact test or χ² test, as indicated by superscript letters. Bold values indicate statistically significant *P* values (2-sided *P* < .05).

a Fisher exact test.

b χ² test.

## Discussion

This secondary analysis is the first, to our knowledge, to compare patient-reported cognitive AEs with clinician-graded cognitive AEs and objective cognitive screening in older adults with advanced cancer receiving systemic therapy. Extending prior work demonstrating that patient-reported outcomes more comprehensively capture symptomatic toxicities,^22^^,^^26^^,^^27^^,^^31^^,^^32^ this study focuses on cognitive symptomatic AEs, an understudied treatment-related toxicity. We also evaluated how these reporting modalities are associated with objective cognitive assessment using Mini-Cog.

Patient-reported cognitive symptomatic AEs were consistently more prevalent than clinician-graded cognitive AEs across all time points. At 4-6 weeks after treatment initiation, 19% of patients reported moderate or greater cognitive symptoms on the Cog-PRO, compared with only 0.48% identified as grade ≥2 by clinician grading. Similar discrepancies were observed at 3 months (18% vs 2.5%) and at 6 months (22% vs 0.44%), suggesting that clinician assessments may underestimate the burden of cognitive symptomatic AEs in this population.^13^ Several factors may explain this discrepancy. Older adults may perceive declines in cognition that are not evident during clinical encounters, whereas treatment-related neurotoxicity, inflammation, fatigue, and emotional distress may further contribute to perceived cognitive impairment.^17^^,^^38^^,^^39^ On the other hand, clinicians may underrecognize cognitive symptoms due to time constraints, competing clinical demands, or the subtle nature of early cognitive changes.^13^^,^^19^^,^^21^^,^^40^ Although the PRO-CTCAE and clinician-rated CTCAE scales both assess AEs, they do so from different perspectives and are not intended to be the same. Instead, the complementary information from the PRO-CTCAE can help investigators better understand the prevalence of symptoms, particularly those of lower grade, and their trajectory over time.^36^^,^^41^^,^^42^ Thus, these measures should be viewed as complementary rather than interchangeable.^42^ This lack of dimensional equivalence may have weakened associations across modalities, particularly between patient-reported symptoms and clinician-graded cognitive AEs, supporting the value of triangulating patient-reported, clinician-graded, and objective cognitive assessments in older adults with cancer.^13^^,^^26^^,^^42^

The marked discrepancy between patient-reported and clinician-graded cognitive symptomatic AEs observed in this study mirrors prior findings in other symptomatic domains, including pain, fatigue, and functional interference, where patient-reported measures consistently identify a substantially higher symptom burden than clinician grading alone.^31^^,^^32^^,^^42^

Our findings extend this pattern to cognitive symptoms, demonstrating persistent under detection despite cognition’s relevance for treatment tolerance, communication, and decision making, and highlighting the substantial cognitive burden during cancer treatment in older adults.^26^^,^^27^^,^^29^^,^^43^^,^^44^

Together, these results are consistent with prior recommendations and underscore the importance of incorporating patient-reported measures alongside clinician grading to more accurately identify and monitor cognitive toxicity in older adults receiving cancer therapy.^26^^,^^29^^,^^30^^,^^45^

In this study, impaired Mini-Cog performance was consistently associated with patient-reported cognitive symptomatic AEs across all time points, supporting the value of objective cognitive screening in oncology care for older adults. However, Mini-Cog impairment and patient-reported symptoms were not fully concordant. Patients with impaired Mini-Cog were more likely to report moderate or greater cognitive symptoms than those without impairment at 4-6 weeks, 3 months, and 6 months: 31% vs 15%, 34% vs 14%, and 49% vs 15%, respectively. This discordance may reflect that Mini-Cog, Cog-PRO, and clinician-graded CTCAE assess related but distinct constructs and perspectives. It may also reflect limited awareness of deficits among some patients with Mini-Cog impairment, cognitive symptoms not captured by this brief screening tool among patients without Mini-Cog impairment, or the influence of mood symptoms on subjective reporting. Together, these findings suggest that Mini-Cog and Cog-PRO are related but not interchangeable, supporting the complementary use of patient-reported symptoms and objective cognitive screening, along with clinician-rated AEs when evaluating cognitive toxicity in older adults with cancer. The Mini-Cog offers a practical advantage as a brief, inexpensive, and validated screening tool that can be readily integrated into routine clinical workflows.^36^^,^^37^^,^^46^ A prior meta-analysis suggests that the Mini-Cog demonstrates a reasonable balance of sensitivity and specificity for detecting cognitive impairment in community-dwelling older adults.^37^ However, its performance in identifying cognitive decline in older adults with cancer remains uncertain and warrants further evaluation using comprehensive neuropsychological benchmarks. Participants with non-impaired Mini-Cog scores were more likely to have some college education or higher than those with impaired Mini-Cog scores, suggesting that education may contribute to baseline differences in Mini-Cog status. However, because the Mini-Cog requires only basic literacy and is commonly used in older adult populations, educational differences may not fully explain the observed impairment.

Despite evidence supporting GA and cognitive screening,^13^^,^^24^^,^^40^ uptake in routine oncology practice remains limited due to time and staffing constraints, particularly for objective assessments requiring trained personnel.^47^ Integrating patient-reported cognitive symptoms with an objective cognitive screening tool alongside clinician grading offers a more holistic approach to detecting clinically meaningful cognitive toxicity, tailoring care for vulnerable older adults during cancer treatment, and identifying patients most likely to benefit from objective screening based on tool-specific properties.^48^

We observed within-patient fluctuation in patient-reported cognitive symptoms over time, indicating that cognitive burden is dynamic during treatment and unlikely to be fully captured by a single assessment.^38^^,^^39^^,^^49^ These findings highlight the importance of repeated, longitudinal cognitive screening rather than reliance on single-time point measures. Prior longitudinal studies in cancer populations similarly show that patient-reported cognitive complaints change over time, with a substantial subset of patients experiencing improvement or decline across follow-up.^17^^,^^38^^,^^39^^,^^49^^,^^50^ Together, these data support incorporating repeated cognitive assessments into routine toxicity monitoring to enable timely identification of emerging or worsening symptoms, more accurate characterization of individual trajectories, and prompt referral for intervention when indicated. Given their vulnerability due to aging, comorbidities, and cancer treatment, enhanced cognitive monitoring is particularly critical for older adults with advanced cancer.^17^^,^^38^^,^^39^^,^^49-51^

Our study has several important clinical implications. Integrating PRO-CTCAE cognitive items into routine oncology care as a rapid, low-burden first-line screening tool may enhance detection and monitoring of cognitive changes, enable timely intervention, and reduce underestimation of symptom burden. Combining PRO-CTCAE with brief objective screening tools such as Mini-Cog may further support early recognition, improve patient-clinician communication, and facilitate shared decision-making.

Key strengths of this study include its large, nationwide, multicenter sample of older adults treated across 40 community oncology practices, longitudinal assessment across multiple time points, and concurrent use of patient-reported, clinician-graded, and objective cognitive measures. The data were derived from the GAP70+ trial,^24^ which incorporated rigorous data verification and centralized review of toxicity reporting. Together, this multi-level approach provides one of the most comprehensive evaluations of cognitive symptomatic AEs in geriatric oncology to date.

This study has several limitations. The cognitive domain of the PRO-CTCAE includes single items for memory and concentration, which may not fully capture the multidimensional nature of cognitive impairment. Similarly, the Mini-Cog, while efficient, has limited sensitivity for subtle deficits. Moreover, Cog-PRO, Cog-CTCAE, and Mini-Cog assess related but distinct cognitive dimensions. These differences may have weakened associations across measures and limit their interpretation as directly comparable. The study cohort was predominantly White and highly educated, which may limit generalizability to other populations. Finally, higher dropout among participants with baseline Mini-Cog impairment may have underrepresented cognitively vulnerable patients at follow-up, potentially leading to underestimation of Mini-Cog impairment and cognitive symptomatic AEs over time. Hence, missing data and attrition over time may have introduced bias.

In conclusion, this study provides novel evidence that patient-reported outcomes capture cognitive symptomatic AEs that are frequently missed by clinician assessments in older adults with advanced cancer, extending prior work demonstrating similar discordance for treatment-related toxicities such as pain and fatigue to the cognitive domain.^22^^,^^31^^,^^32^ Integrating patient-reported cognitive outcomes alongside clinician-graded AEs and brief objective screening offers a more comprehensive, patient-centered approach to assessing treatment-related cognitive toxicity and improving quality of care in this population. Future research should expand the use of PRO-CTCAE cognitive items in observational and interventional studies, evaluate their predictive value for treatment adherence, functional outcomes, and survival, and examine how integration with neuropsychological assessments, biologic correlates, and routine clinical workflows can further advance understanding and implementation in oncology practice.^13^

## Supplementary Material

pkag086_Supplementary_Data

## Data Availability

The data underlying this article derive from a secondary analysis of clinical trial data and cannot be shared publicly due to patient privacy and institutional restrictions. De-identified data may be made available on reasonable request to the corresponding author, subject to appropriate institutional approvals and data use agreements.
