# Supplementary material for: Prevalence of patient-reported and clinician-graded cognitive symptomatic adverse events in older patients with advanced cancer
Source: JNCI Cancer Spectr. 2026 Aug 24;10(5):pkag086. doi: 10.1093/jncics/pkag086 (PMC13600430; doi:10.1093/jncics/pkag086)
Supplement: Supplementary file 1 [file pkag086_supplementary_data.zip › Clean Revised_#2_Final Supplementary Tables.docx]

**Supplementary Material**

**Table S1. Frequency and Prevalence of Cognitive Measures Across Assessment Timepoints**

| **Assessment Timepoint** | **Cognitive Measures** | | |
| --- | --- | --- | --- |
|  | **Cog-CTCAE (Grade ≥ 2)** | **Cog-PRO (Moderate or Greater)** | **Mini-Cog Impairment** |
| **A1 (Baseline)** | (Not collected) | **No:** 574 (81%) | **No:** 461 (64%) |
|  |  | **Yes:** 131 (19%)  **95% CI**: 16%, 22% | **Yes:** 257 (36%)  **95% CI:** 32%, 39% |
| **A2 (****4-6 weeks)** | **No:** 618 (99%) | **No:** 505 (81%) | **No:** 480 (79%) |
|  | **Yes:** 6 (0.96%)  **95% CI:** 0.35%, 2.08% | **Yes:** 116 (19%)  **95% CI:** 16%, 22% | **Yes:** 120 (20%)  **95% CI:** 17%, 23% |
| **A3 (3 months)** | **No:** 516 (98%) | **No:** 429 (82%) | **No:** 414 (80%) |
|  | **Yes:** 13 (2.46%)  **95% CI:** 1.31%, 4.17% | **Yes:** 92 (18%)  **95% CI:** 14%, 21% | **Yes:** 95 (19%)  **95% CI:** 15%, 22% |
| **A4 (6 months)** | **No:** 451 (99.56%) | **No:** 351 (78%) | **No:** 345 (80%) |
|  | **Yes:** 2 (0.44%)  **95% CI:** 0.05-1.59% | **Yes:** 100 (22%)  **95% CI:** 18%, 26% | **Yes:** 79 (18%)  **95% CI:** 15%, 23% |

Notes:

- Cog-CTCAE = clinician-graded cognitive symptoms (No: grade ≤ 1; Yes: grade ≥ 2)
- Cog-PRO = patient-reported cognitive symptoms (moderate or worse severity/interference)
- Mini-Cog = objective cognitive impairment screening
- CI = 95% exact confidence interval
- 95% confidence intervals correspond to the prevalence of grade ≥2 (Yes) cognitive adverse events.
- CTCAE not collected at baseline (A1)

**Table S2. Frequency and proportion/prevalence of Mini-cog impairment at A1, A2, A3, and A4, respectively**

|  | **A1** | | | | **A2** | | | **A3** | | | **A4** | | |
| --- | --- | --- | --- | --- | --- | --- | --- | --- | --- | --- | --- | --- | --- |
| **Mini-cog impairment** | **N** | **%** | **Valid %** | **N** | | **%** | **Valid %** | **N** | **%** | **Valid %** | **N** | **%** | **Valid %** |
| Non-Impaired | 461 | 64 | 64 | 480 | | 79 | 80.00 | 414 | 80 | 81 | 345 | 80 | 81 |
| Impaired | 257 | 36 | 36 | 120 | | 20 | 20.00 | 95 | 18 | 19 | 79 | 18 | 19 |
| Missing | 0 | 0.00 |  | 8 | | 1.32 |  | 6 | 1.1 |  | 8 | 1.9 |  |
| **95% exact CI** for the proportion/prevalence of Mini-cog impairment |  |  | (32%, 39%) |  | |  | (17%, 23%) |  |  | (15%, 22%) |  |  | (15%, 23%) |

CI: confidence interval

**Table S3. Changes in Patient-Reported Cognitive Symptoms (Cog-PRO) Across Assessment Timepoints**

|  |  |  | **Adjustment for Multiple Comparisons** | |
| --- | --- | --- | --- | --- |
|  |  |  | **P-values** | |
|  | **OR (95% CI)** | **P-value** | **Raw** | **Sidak** |
| **Omnibus test for change of Cog-PRO*** **over four assessments** |  | **0.004** |  |  |
| Change of Cog-PRO from A1 to A2 (A2 minus A1) | 1.03 (0.83, 1.28) |  | 0.776 | 0.999 |
| Change of Cog-PRO from A1 to A3 (A3 minus A1) | 1.05 (0.85, 1.29) |  | 0.670 | 0.9987 |
| Change of Cog-PRO from A1 to A4 (A4 minus A1) | 1.42 (1.14, 1.77) |  | 0.002 | **0.011** |
| Change of Cog-PRO from A2 to A3 (A3 minus A2) | 1.01 (0.83, 1.24) |  | 0.885 | 1.000 |
| Change of Cog-PRO from A2 to A4 (A4 minus A2) | 1.38 (1.10, 1.72) |  | 0.005 | **0.028** |
| Change of Cog-PRO from A3 to A4 (A4 minus A3) | 1.36 (1.11, 1.66) |  | 0.003 | **0.018** |

Results of crude binary logistic GEE model analysis for *Pro-CTCAE_1* (patient-reported cognitive symptoms; No vs. Yes) across assessment timepoints (A1 = baseline, A2 = 4–6 weeks, A3 = 3 months, A4 = 6 months). Odds ratios (OR) and 95% confidence intervals (CI) represent the relative odds of reporting cognitive symptoms at later assessments compared with earlier timepoints. P-values were adjusted for multiple comparisons using the Sidak correction.

*Cog-PRO: Patient-reported cognitive symptoms.

**Table S4. Baseline Geriatric Assessment Domains and Measures**

| **Measure** | **Overall**  **(N=704)** | **Impaired mini-cog**  **(n=248)** | **Normal mini-cog (n=456)** | **p-value** |
| --- | --- | --- | --- | --- |
| **Impaired Activity of Daily Living** |  |  |  | **0.05** ^a^ |
| No | 514 (73) | 170 (69) | 344 (75) |  |
| Yes | 190 (27) | 78 (31) | 112 (25) |  |
| **Impaired Medical Social Support** |  |  |  | 0.7 ^a^ |
| No | 514 (73) | 179 (72) | 335 (73) |  |
| Yes | 190 (27) | 69 (28) | 121 (27) |  |
| **Impaired Generalized Anxiety Disorder** |  |  |  | **0.009** ^a^ |
| No | 616 (88) | 206 (83) | 410 (90) |  |
| Yes | 88 (13) | 42 (17) | 46 (10) |  |
| **Impaired mini nutritional assessment** |  |  |  | 0.4 ^a^ |
| No | 287 (41) | 96 (39) | 191 (42) |  |
| Yes | 417 (60) | 152 (61) | 265 (58) |  |
| **Impaired Physical Health** |  |  |  | **0.01** ^a^ |
| No | 175 (25) | 48 (19) | 127 (28) |  |
| Yes | 529 (75) | 200 (81) | 329 (72) |  |
| **Impaired Falls** |  |  |  | **0.02** ^a^ |
| No | 555 (79) | 183 (74) | 372 (82) |  |
| Yes | 149 (21) | 65 (26) | 84 (18) |  |
| **Impaired Geriatric Depression Scale** |  |  |  | **<0.001** ^a^ |
| No | 540 (77) | 170 (69) | 370 (81) |  |
| Yes | 164 (23) | 78 (31) | 86 (19) |  |
| **Impaired Comorbidity** |  |  |  | **0.02** ^a^ |
| No | 229 (33) | 67 (27) | 162 (36) |  |
| Yes | 475 (67) | 181 (73) | 294 (64) |  |
| **Impaired Polypharmacy** |  |  |  | 0.3 ^a^ |
| No | 133 (19) | 42 (17) | 91 (20) |  |
| Yes | 571 (81) | 206 (83) | 365 (80) |  |

^a^Chi-square test; ^b^ Wilcoxon rank-sum test
